# Case Report: Circulating Tumor Cells as a Response Biomarker in ALK-Positive Metastatic Inflammatory Myofibroblastic Tumor

**DOI:** 10.3389/fped.2021.652583

**Published:** 2021-04-29

**Authors:** Paolo Bonvini, Elisabetta Rossi, Angelica Zin, Mariangela Manicone, Riccardo Vidotto, Antonella Facchinetti, Lucia Tombolan, Maria Carmen Affinita, Luisa Santoro, Rita Zamarchi, Gianni Bisogno

**Affiliations:** ^1^Institute of Pediatric Research, Fondazione Città della Speranza, Padova, Italy; ^2^Department of Surgery, Oncology and Gastroenterology, Oncology Section, University of Padova, Padova, Italy; ^3^Veneto Institute of Oncology IOV - IRCCS, Padova, Italy; ^4^Hematology and Oncology Unit, Department of Women's and Children's Health, University of Padova, Padova, Italy; ^5^Department of Medicine, Padova University Hospital, Padova, Italy

**Keywords:** IMT, ALK, CTC, metastasis, CellSearch, liquid biopsy

## Abstract

Inflammatory myofibroblastic tumors (IMTs) are locally aggressive malignancies occurring at various sites. Surgery is the mainstay of treatment and prognosis is generally good. For children with unresectable or metastatic tumors, however, outcome is particularly severe, limited also by the lack of predictive biomarkers of therapy efficacy and disease progression. Blood represents a minimally invasive source of cancer biomarkers for real-time assessment of tumor growth, particularly when it involves the analysis of circulating tumor cells (CTC). As CTCs potentially represent disseminated disease, their detection in the blood correlates with the presence of metastatic lesions and may reflect tumor response to treatment. Herein, we present a case report of a 19-year-old boy with an ALK-positive IMT of the bladder, proximal osteolytic and multiple bilateral lung lesions, who received ALK inhibitor entrectinib postoperatively and underwent longitudinal CTC analysis during treatment. Antitumor activity of entrectinib was demonstrated and was accompanied by regression of lung lesions, elimination of CTCs from the blood and no development of relapses afterwards. Therapy continued without any clinical sign of progression and 24 months since the initiation of treatment the patient remains symptom-free and disease-free.

## Introduction

Inflammatory myofibroblastic tumor (IMTs) is a rare low-grade neoplasm of childhood and adolescence, occurring mainly in the lung and liver, and less frequently at other sites, including the abdominal cavity (1). Histologically, IMTs consist of spindle-shaped myofibroblastic elements with accompanying inflammatory infiltrates of plasma cells and lymphocytes, whereas at a molecular level fusions involving the anaplastic lymphoma kinase (*ALK*) gene, and less frequently *ROS1, NTRK1, PDGFRB, and RET*, are found in at least half of cases and result in the overexpression and constitutive activity of the correspondent protein tyrosine kinase (2, 3). Surgery remains the treatment of choice, but incomplete resection frequently associates with local relapse or metastasis (4). Patients with unresectable or recurring disease have few treatment options, including the ALK inhibitor crizotinib (5, 6) or second-generation kinase inhibitors, which are used when resistance to crizotinib develops and disease progresses (7–9). In consideration of the dismal prognosis of patients with relapsed/refractory IMTs, predictive biomarkers of therapy efficacy are needed, preferably if obtained with minimally invasive, low risky and repeatable approaches. Liquid biopsies have recently become important as an alternative to surgical biopsies and source of tumor markers, both for the purpose of monitoring disease spreading and response to treatment throughout the analysis of circulating tumor cells (CTCs) or other soluble factors (10). In particular, clinical evidence indicates that patients with metastatic lesions are more likely to have CTCs in the blood, while in patients with localized disease CTCs are extremely rare or even absent. Longer progression-free survival rate is observed in cancer patients where CTC count drops with therapy, while relapses frequently occur when CTCs remain detectable or even increase during treatment (11). Liquid biopsy is a minimally invasive source of tumor cells over the course of the disease, particularly important when tumor material is not available or when classical biopsy may cause risks to patients. So far, few studies focusing on the value of CTCs level as surrogate marker of therapy effectiveness have been performed and none of them have involved pediatric patients. Herein, we report a case of a boy with ALK-positive metastatic urinary bladder IMT successfully treated with the ALK inhibitor entrectinib and monitored longitudinally for the presence of CTCs in the peripheral blood. We provided the evidence that CTCs can be detected at diagnosis, and changes in their level over the course of treatment correlate with the patient's clinical condition and final outcome.

## Case Presentation

**Figure 1:**
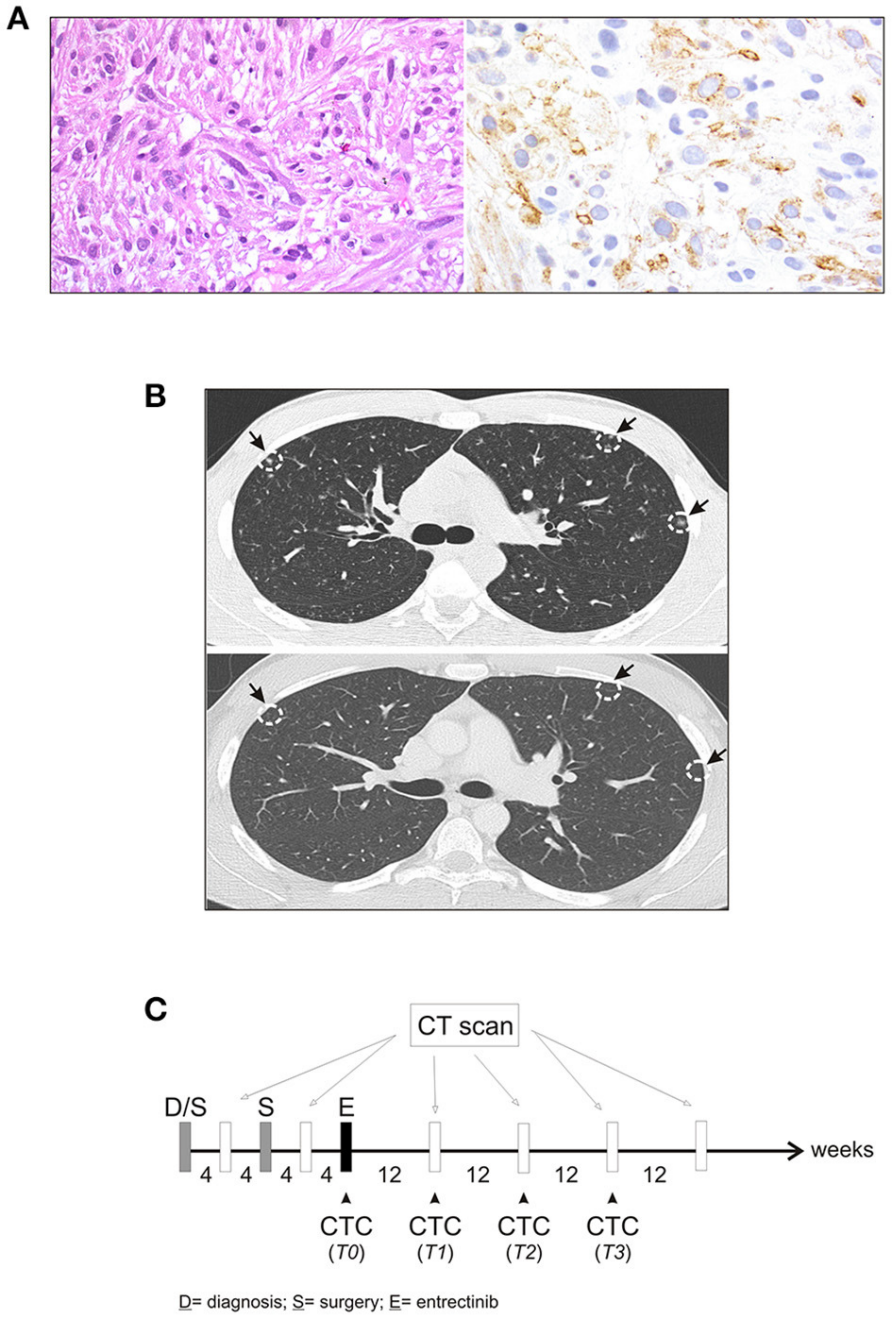
Characteristics of IMT. **(A)** Fascicular proliferation of monotonous spindle cells within myxoid stroma (*left*), and immunohistochemical staining with anti-ALK antibody (*right*). **(B)** Computed tomography (CT) scan before initiating entrectinib (*top*), and after 9 months of entrectinib (*bottom*). Arrows indicate lung micronodules. **(C)** Workflow for CT scan and longitudinal CTCs collection during treatment.

In January 2017, a 19-year-old boy came to our attention with macroscopic hematuria and progressive anemia. The patient reported mild asthenia, with no other systemic symptoms. Blood investigation including inflammatory test (CRP and procalcitonin) were normal. Sonography identified a mass that, on abdominal magnetic resonance imaging (MRI), appeared as a locally thickened and edematous mucosa of the anterior wall of the bladder. A transurethral resection was performed at the local hospital. The patient arrived to our center 7 weeks later and an endoscopic evaluation was performed with no evidence of a residual mass. The histology of the tumor initially resected was reviewed revealing an ALK-positive IMT. The tumor was characterized by spindle cells with ovoid nuclei containing prominent nucleoli and eosinophilic cytoplasm. The tumor cells were interspersed in a myxoid stroma with a heterogeneous inflammatory infiltrate (Figure 1A, *left*). Immunohistochemistry was positive in more than 50% of cells for classical IMT markers, such as ALK, alpha smooth muscle actin and desmin, and there was focal expression of cytokeratins (CK) 8/18/19. Positivity for ALK varied, with cells showing more or less diffuse cytoplasm staining (Figure 1A, *right*). On staging, CT scanning revealed multiple small nodules in both lungs, mainly in the upper left lobe (Figure 1B, *top*), and 18 fluorodeoxyglucose-PET showed a 29 × 18 × 29 mm osteolytic lesion in the left iliac bone, subsequently confirmed by bone CT scan. A diagnosis of metastatic ALK-positive IMT was made and an anti-ALK therapy (entrectinib 600 mg/day) was adopted (Figure 1C). Treatment response was assessed after 3 months, indicating stable disease. As CT images confirmed a stable condition in terms of the number and size of lung and bone metastases, entrectinib administration was continued. Three months later, CT scanning revealed excavated pulmonary micronodules suggestive of initial drug response, whereas after 9 months a substantial reduction of the nodules, in particular the excavated pulmonary micronodules in the right lung, and a persistent bone metastasis was observed. One year after starting the therapy all lung metastases disappeared (Figure 1B, *bottom*) and the bone lesion was much reduced in size (23 vs. 29 mm), showing sclerotic margins around the central lytic area. During the treatment the patient was followed monthly for the 1st year with progressively longer intervals in the 2nd year. No side effects were reported apart a mild episode of diarrhea in the 2nd month of treatment. Blood investigations including blood count, renal and liver function and CRP were always normal. Due to the favorable safety profile of entrectinib, the therapy continued and 33 months after starting the treatment the patient remains in complete remission.

**Figure 2:**
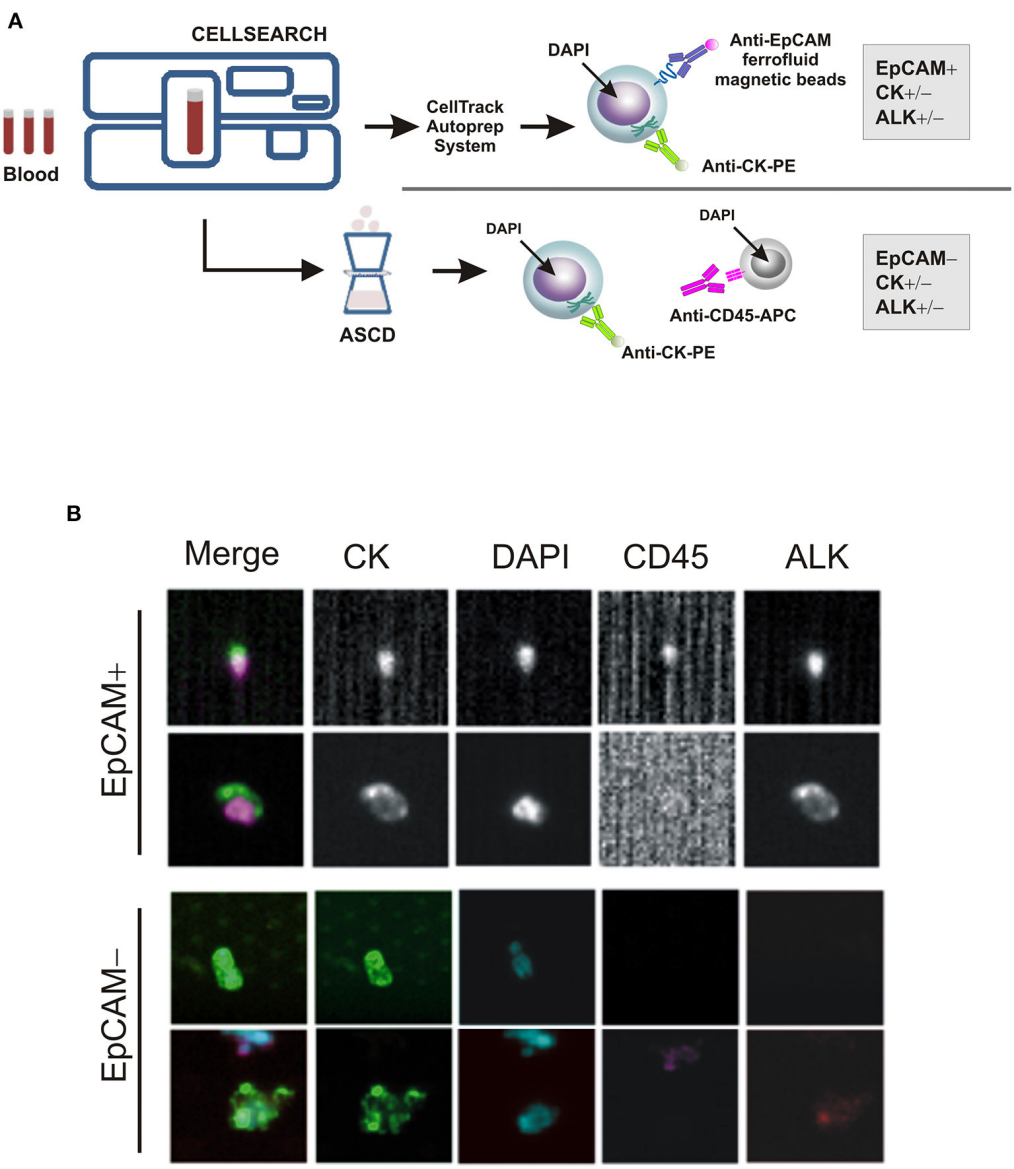
CTC enrichment and detection. **(A)** The CTC enrichment approach used to detect and isolate EpCAM-positive (EpCAM^high/+^) and negative (EpCAM^low/−^) CTCs from cancer patients' blood samples by the CellSearch system and the ASCD device, respectively. APC-tagged anti-CD45 antibody is used to detect and distinguish peripheral blood leukocytes from CTCs, whereas ferrofluid-tagged and PE-tagged anti-EpCAM and -CK antibodies to isolate EpCAM^+^/CD45^−^ and/or CK^+^/CD45^−^ cells, respectively. **(B)** Representative images of EpCAM^+^ and EpCAM^−^ CTCs captured by CellSearch and ASCD, and stained for cytokeratins (CK), CD45, ALK or with nuclear dye DAPI.

As a part of an ongoing study on sarcomas, we tested for the presence of CTCs in patient's whole blood samples, prior to and throughout the treatment, using the CellSearch^TM^ system (CS): the only EpCAM-based CTC enrichment system approved by the FDA (Food and Drug Administration) for the *In vitro* Diagnostic (IVD) detection and collection of circulating tumor cells in cancer patients (12). According to the system, an event is classified as CTC when its morphological features are consistent with that of a cell expressing the epithelial cell adhesion molecule (EpCAM^high/+^) and exhibiting a CK^+^, DAPI^+^ and CD45^−^ phenotype (Figure 2A). However, since EpCAM expression may change during tumor evolution and CTC expressing low levels of EpCAM are discarded by the CellSearch system, cells expressing no or low EpCAM (EpCAM^low/−^) were also collected through an antibody-independent Automatic Sample Collection Device (ASCD) coupled to our CellSearch system and subsequently stained for CK, CD45, and DAPI (Figure 2A). Finally, to capture circulating ALK-positive IMT cells, a customized assay with a FITC-conjugated anti-ALK antibody was designed (13) and results were expressed as the total number of ALK^+^CTCs per 7.5 ml of peripheral blood (Figure 2B). As summarized in Table 1, which reports findings obtained through liquid biopsy, EpCAM^high/+^ cells were found in the blood before starting entrectinib administration (Table 1, *T0*), with one in two of them being ALK^−^, whereas no cells were detected at the end of the first treatment cycle when the disease was reportedly stable (Table 1, *T1*). The 6-month assessment revealed 2 ALK^+^/CK^+^/CD45^−^ and 2 ALK^−^/CK^+^/CD45^−^ circulating tumor cells (Table 1, *T2*). EpCAM was variably expressed in the 2 ALK^+^ cells (1 EpCAM^high/+^ and 1 EpCAM^low/−^), whereas both of them were positive to CK, negative to CD45 and had intact nuclei stained by DAPI. At this time, concomitant CT images revealed persistent focal pulmonary ground-glass opacities with excavated micronodules of branching “tree-in-bud” morphology, suggesting a partial response to entrectinib. After 9 months of targeted therapy, no more circulating IMT cells were detected and this situation remained the same up to the last follow-up (Table 1, *T3*). This last CTCs assessment was performed prior to CT images of complete pulmonary metastasis reduction, hence earlier than clinical response. Since then, our patient exhibited a continuous improvement in symptoms over the time and no additional adverse events were reported.

**Table 1:** Patient's CTC counts by CellSearch (EpCAM^high/+^) and ASCD (EpCAM^low/−^) system.

	T0^*^		**T1**		**T2**		**T3**	
	**Phenotype**	No. CTCs^**^	**Phenotype**	No. CTCs^**^	**Phenotype**	No. CTCs^**^	**Phenotype**	No. CTCs^**^
**CellSearch (EpCAM**^**high/+**^**)**	EpCAM+ CK+ ALK+	1	EpCAM+	0	EpCAM+ CK+ ALK+	1	EpCAM+	0
	EpCAM+ CK+ ALK-	1	/	/	/	/	/	/
**ASCD (EpCAM**^**low/−**^**)**	/	/	EpCAM-	0	EpCAM- CK+ ALK+	1	EpCAM-	0
	/	/	/	/	EpCAM- CK+ ALK-	2	/	/
	**All**	**2**	**All**	**0**	**All**	**4**	**All**	**0**
**Therapy timeline**		Before starting entrectenib		End of the 1st treatment cycle		6-months re-evaluation		9-months re-evaluation
**Imaging**		**CT/PET**		**CT**		**CT**		**CT**
**Report**		Multiple small nodules in both lungs; Osteolytic lesion in the left iliac bone		Stable number and size of lung and bone metastasis		Pulmonary opacities		Complete reduction of lung metastasis
**Objective response**		***–***		**SD^§^**		**PR**		**CR**

* *T0: blood withdrawal at baseline, T1–T3: blood withdrawal during therapy (T = time-point)*.

** *CTCs levels are expressed as number of CTCs per 7, 5 mL peripheral blood*.

§ *SD = Stable disease; PR = Partial response; CR = Complete remission*.

## Discussion

Targeting the *ALK* gene in childhood cancer is particularly effective in ALK fusion-positive tumors, as genomic alterations leading to the expression of truncated variants render ALK kinase constitutively active and cancer cells addicted to it. A strong and durable clinical activity of the ALK inhibitor crizotinib has been demonstrated in fusion-positive anaplastic large cell lymphomas and IMT tumors, whereas its efficacy has been limited in malignancies expressing full-length ALK kinase. In IMT patients, crizotinib has been used as first-line therapy or after surgery, with an overall response rate of 67 and 86%, respectively, a median duration of therapy varying between 9 months and 1.67 years, and responses lasting to over 2 years after treatment discontinuation (14, 15). The National Comprehensive Cancer Network guidelines recommend using ALK inhibitors for IMT patients with *ALK* gene fusions, though there is no targeted agent specifically approved. Entrectinib is a tyrosine kinase inhibitor currently being tested in phase I/II adult and pediatric clinical trials for patients with solid tumors harboring *NTRK, ROS1*, or *ALK* gene fusions. Two pediatric IMT patients with *ROS1* and *ALK* rearrangements have been treated with entrectinib after surgery, achieving a complete response at the end of their treatment (16). Neither, however, presented with metastases at diagnosis.

Herein, we used entrectinib successfully in a patient with metastatic ALK-positive IMT of the bladder and reported the detection of IMT circulating tumor cells from whole blood collected at a random time during therapy. To our knowledge, no IMT of the bladder with distal metastases have been treated with entrectinib so far, and no IMT patient has ever been monitored using CTCs as a marker of therapeutic response. We obtained the evidence that CTCs can be detected at baseline and monitored during treatment with entrectinib, using a quantitative approach customized for both EpCAM and ALK expression: two markers previously used to detect CTCs of both epithelial and mesenchymal origin. The number of CTCs changed over the course of the treatment and paralleled the patient's radiological response and clinical condition. CTCs were detected when partial disease control was achieved, whereas they became undetectable at patient's complete remission. As for the nature of these cells, both EpCAM and ALK expression varied during the longitudinal sample collection, while CK expression remained constant. In line with our findings, IMTs of the bladder positive for cytokeratins have been described, likewise ALK-negative IMT tumors successfully treated with small-molecule kinase inhibitors because turned out to be ALK-positive when reassessed with more sensitive detection tools (17–20). Previous reports have also shown that epithelial and mesenchymal CTCs expressing no or low EpCAM are detected in specific tumor types and associated with distal metastasis and worse prognosis (21–24). As the coexistence of EpCAM^high/+^ and EpCAM^low/−^ tumor cells is likely, the heterogeneity of CTCs must be taken into consideration when monitoring therapeutic response to treatment and disease progression in individual patients, as we did here.

With the goal of developing and evaluating CTC-based diagnostic and prognostics for pediatric solid tumors, we made similar observations in children with tumor types other than IMT. In patients with renal cell carcinoma (RCC) we demonstrated that the enumeration of CTCs is actionable during targeted therapy and correlates with patients' clinical conditions and final outcome (negative at complete remission, whilst positive over the follow-up period in patients experienced relapse) (25). In children with soft tissue and bone sarcomas, such as rhabdomyosarcoma, Ewing sarcoma and osteosarcoma, the higher was the expression of EpCAM and the number of CTCs at diagnosis the worse was the prognosis (26).

We were aware that our study shows some limits, implicit of case reporting studies, namely one patient, one treatment, and limited follow-up and timeline schedule of blood draws, which finally do not allow generalizing study' findings.

However, these findings offer the rationale to further investigations of CTCs in larger pediatric cohorts, since we demonstrated here, for the first time, the feasibility of using the ALK-integrated CTC test in a malignancy different from that for which it has been developed originally (i.e., adult NSCLC).

Overall, our study proves the effectiveness of entrectinib in the treatment of a patient with a metastatic IMT at diagnosis and the feasibility of longitudinal CTC analysis for non-invasive disease monitoring during treatment. A future, larger study is needed to investigate whether serially collected CTCs are also potential predictive biomarkers of disease recurrence. It might represent a turning point in understanding the evolution of solid tumors like IMT during treatment and progression, as this phenomenon in children and young patients is dramatically fast and hopelessly. There is no doubt, however, that liquid biopsies will provide in the next future pediatric oncologists with a much better view of disease dissemination and individual patient's response to treatment, allowing therapy optimization, personalization and, ultimately, leading to a better outcome.

## Data Availability Statement

Any data and materials are available from the corresponding author on reasonable request.

## Ethics Statement

The studies involving human participants were reviewed and approved by Ethics Committee for Clinical Trials of the Province of Padua. The patients/participants provided their written informed consent to participate in this study.

## Author Contributions

PB, ER, RZ, and GB conceived and designed the study. AZ, ER, LT, MM, RV, and AF performed the experiments. LS provided the IHC results. MCA and GB were responsible for patient's management and collection of clinical data. PB wrote the paper. ER, RZ, and GB reviewed and edited the manuscript. All authors read and approved the manuscript.

## Conflict of Interest

The authors declare that the research was conducted in the absence of any commercial or financial relationships that could be construed as a potential conflict of interest.

## Abbreviations

ALK: anaplastic lymphoma kinase
ASCD: autoprep sample collection device
CK: cytokeratins
CS: CellSearch
CT: computed tomography
CTC: circulating tumor cells
EpCAM: epithelial cell adhesion molecule
IMT: inflammatory myofibroblastic tumor
MRI: magnetic resonance imaging.
